# Growth of Plasma-Treated Corn Seeds under Realistic Conditions

**DOI:** 10.1038/s41598-019-40700-9

**Published:** 2019-03-13

**Authors:** Chisung Ahn, John Gill, David N. Ruzic

**Affiliations:** 1 Center for Plasma Material Interactions, Department of Nuclear, Plasma and Radiological Engineering, University of Illinois at Urbana-Champaign, Urbana, IL 61801 USA; 2 AgReliant Genetics, LLC., Ivesdale, IL 61851 USA

## Abstract

In this study, the effect of the plasma treatment on corn seeds is investigated. Corn seeds were treated uniformly without burning or blackening by three kinds of plasma apparatus: RF plasma in vacuum, microwave-driven atmospheric-pressure plasma, DBD atmospheric-pressure plasma, and two other treatments: vacuum exposure only, and using plasma-activated water in the seed coating process, to investigate growth rate changes under realistic conditions. Each treatment was performed on a total of 1512 corn seeds. Seeds from each experimental condition were treated with the recommended rate of Poncho/VOTiVO with Acceleron, a commercial biological seed treatment that helps to protect the seeds from fungus, insects, and nematodes after planting. The 1512 seeds were divided evenly into three replications with 84 seeds planted for each replication at six unique locations across central Illinois. The results for germination, growth, and product yield over the 2017 growing season is presented. Overall no statistically significant difference in the yield of corn harvested was found between the control and any of the five treatments. This is likely due to the already near-100% germination rate of the corn hybrid used in the study and the use of the Poncho/VOTiVO protective coating on every sample.

## Introduction

About 35% of the world corn production now is harvested^1^ from more than 80 million acres (about 323,749 km^2^) in the United States, mostly in Illinois, Iowa and Indiana^2^. Most of this corn is used in livestock feed, but it also is processed into a multitude of food and industrial products such as high-fructose corn syrup, starch, cereal, sweetener, beverages, alcohol and ethanol as fuel respectively^3^. There is also a significant export market. This means the corn harvest rate influences the economy not only in the US but also globally, therefore increased yield, even by a small fraction, is highly desired. For this reason, farmers and seed researchers strive to demonstrate increased volume of corn kernels produced per land area.

Recently, plasma treatment technology has been focused on a practical way to improve the yield of agricultural seeds in large quantities because it has demonstrated elimination of unwanted microbes, water absorption control, introduction of functional groups^4–8^ or other economic and environmental effects^9^. Especially, cold-plasma treatment as a non-thermal technology is desirable for enhanced germination and growth-rate of many kinds of crops by disinfection and surface modification. For example, almonds can be decontaminated by surface plasma exposure^10^ and short plasma treatment time of wheat seeds have shown noteworthy effect on improvement of germination and growth rate by significant reduction of epiphytic bacteria and phytopathogenic and toxicogenic filamentous fungi^11^. Also, with a plasma-activated medium which has nitrogen as its main component one can affect the efficiency of water used in the germination phase^12,13^. Meanwhile, hybrid corn in the US has been developed extensively with nearly perfect germination rates but still is subject to soil borne pests and fungus. To find out if there is an effect from plasma treatments on hybrid corn seeds, as seen in the lab environment done by other groups^8,9,14^, it is required to investigate a variety of seed treatments on an industrial agriculture scale and measure actual crop yields.

The work from other groups show that plasma-related treatments can have a positive effect on speed of germination, plant heights, early vigor, growth rate, etc. These markers are expected to have a positive effect on yield at the end of the year since the corn plants would be exposed to sunlight sooner and have a longer growing season available. Our experiment was designed to measure the one thing which is the most important: the yield of crop per unit area under realistic conditions. To compare the various plasma conditions, six experimental treatments were evaluated: a control group, Plasma Activated Water (PAW) treatment of the seed coating material, Microwave Atmospheric Plasma (MAP) on the seeds themselves, atmospheric pressure Dielectric Barrier Discharge (DBD) plasma on the seeds, low pressure Radio Frequency (RF) plasma on the seeds and just exposure of the seeds to vacuum. The results of product yield over the 2017 growing season in six separate locations of Illinois is presented here.

## Methods

### Quantization of corn seed before plasma treatment

The seed utilized for the experiment is a yellow dent corn hybrid adapted to the US Midwest. There are approximately 3.3 kernels per 1 gram, so an electronic balance was used to count the corn seeds before treatment. Through this way, we treated approximately 1500 seeds for each of five plasma conditions along with 1500 control seeds and planted about 300 kernels from each group in each of six different locations.

### Plasma apparatuses for corn treatments

#### Microwave atmospheric plasma (MAP) jet

**Figure 1:**
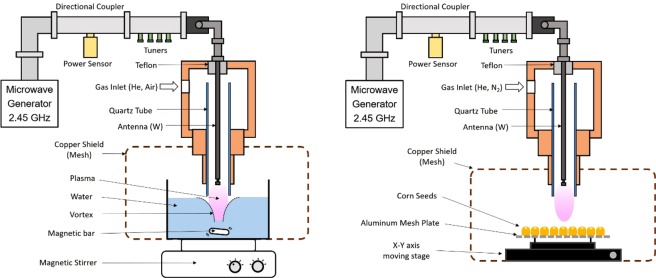
(**a**) Schematic of microwave atmospheric pressure plasma (MAP) system to make plasma-activated water (PAW). (**b**) Schematic of MAP system used to treat corn seeds.

The MAP jet system was designed and fabricated at the Center for Plasma Material Interactions (CPMI) at University of Illinois at Urbana-Champaign (UIUC) for generating a plasma jet^15,16^. The plasma activated water (PAW) was produced by ionic species of a Helium-Air plasma which is generated by the MAP jet as shown in Fig. 1a. Normally, PAW has improved germination of plants by creating a higher concentration of nitrate (NO^3−^) as an essential nutrient^12,17^. To make PAW, 800 W of microwave power was deposited in a mixture of gases consisting of 10 LPM of helium and 5 LPM of air for 10 minutes. To improve the absorption of ions in the water a vortex was initiated by spinning a magnetic bar in the water. With this process, 1.5 liters of wat)er with 40 ppm of NO^3−^ was produced as measured by indicator strips (LaMotte Co, USA). Hydrogen peroxide was looked for and seen only near the detectability threshold, less than 0.5 ppm. In addition the pH of the water was not significantly depressed. Then PAW was mixed with Poncho/ VOTiVO with Acceleron (a powdered biological treatment) as opposed to tap water. The creation of the PAW, mixing of the powder and application of the biological treatment was conducted immediately before planting the corn seed in the field. This was done to see if the effect of PAW on disinfecting microbes and fungi during growth would translate into this process. However, very little water – approximately 1 ml–is used in the process of coating an entire group of 1500 seeds, so direct comparison to using PAW as the primary water source will be difficult.

Corn seeds treated by a direct He-N_2_ plasma generated by a MAP jet can be seen in Fig. 1b. Treatment conditions were 500 W of microwave power, using a mixture of gas with 15 LPM of helium and 3 LPM of N_2_, for a 3 second plasma exposure time. The diameter of plasma was about 2 cm and velocity of moving stage was 0.7 cm/seconds which resulted in about 3 seconds of plasma exposure time for each corn seed. Each time, about 120 kernels were treated and eventually, all 1512 corn kernels were exposed. The 3 second exposure time was chosen to prevent seeds from visibly turning brown or burning. However, the gas temperature of the MAP jet is on the order of 600 °C or higher so some heating of the seeds is likely.

#### Dielectric-barrier discharge (DBD) plasma

**Figure 2:**
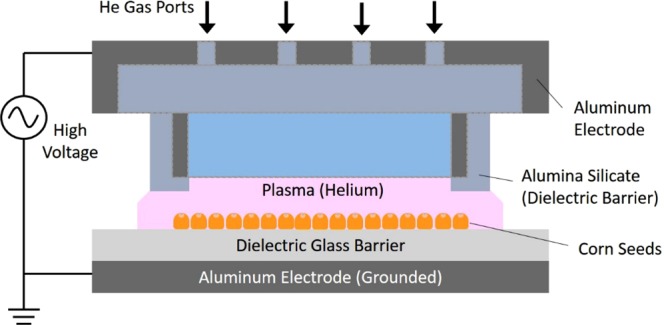
Dielectric-barrier discharge (DBD) system used to treat corn seeds with a helium plasma.

The DBD plasma was also designed and fabricated at the CPMI at the UIUC as shown in Fig. 2 and used for treatment of corn seeds at lower temperature at atmospheric pressure. 10 LPM of helium was used as the process gas and the plasma discharge was maintained between a glass plate and the electrode with a gap of 1 cm by applying a high voltage of 15 kV at 35 kHz. The plasma exposure area was about 2 cm in width by 10 cm long. The corn seeds were distributed in one layer on a 10 cm by 10 cm plate. The tray was moved 2 cm by hand five times to cover the entire 10 cm tray every 10 seconds, resulting in 10 seconds of plasma exposure time for any given seed. The plasma characteristics were very similar to those shown in the paper by Y. Wu^18^. Treatment time was again designed to not burn or brown the seeds. The energy density of this treatment is lower than MAP and does not produce high gas temperatures though the temperature is slightly higher (~ 5 to 10 C) than room temperature.

#### Low pressure radio frequency (RF) plasma

**Figure 3:**
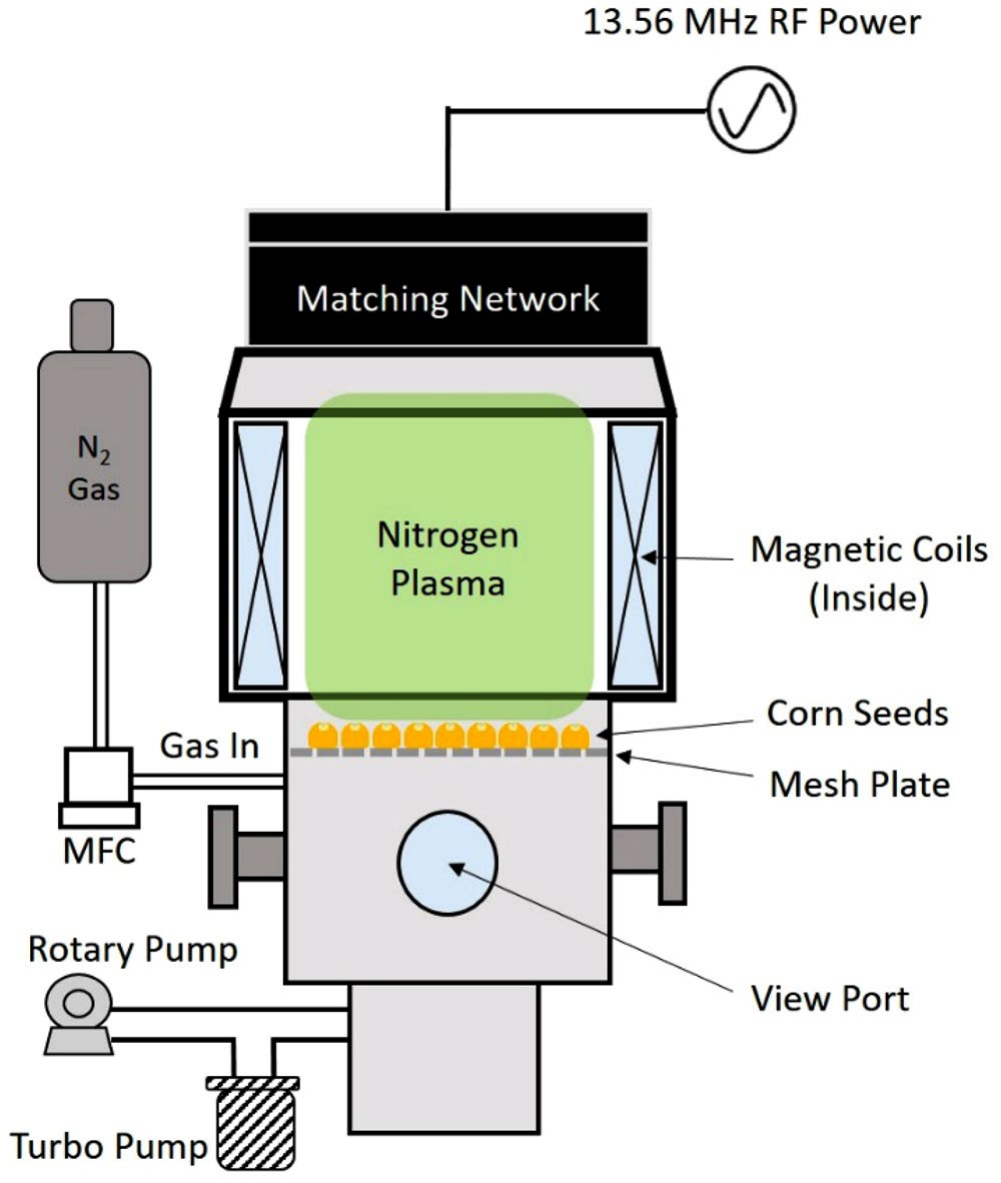
13.56 MHz radio-frequency (RF) plasma system utilizing a helicon used to treat corn seed with a low-pressure plasma and the same system which produced the vacuum-only procedure.

The RF (13.56 MHz) plasma system shown in Fig. 3 was also designed and fabricated at the CPMI at the UIUC^19^ and was used to treat the corn seeds under a low-pressure plasma condition. To generate a low-pressure plasma, a helicon plasma source was employed and N_2_ gas was used as process gas. About 1500 corn seeds were placed on a mesh structure which was installed in the chamber, and the chamber was taken to its base pressure of 10^−6^ Torr in 9 minutes. Nitrogen gas was then added quickly raising the pressure to 100 mTorr. A power of 800 W was then applied creating the plasma for 2 minutes. Another set of 1500 corn seeds were treated under the vacuum condition only at 10^−6^ torr for a total time of 10 minutes including the pump-out as a control to see if the vacuum treatment alone would have an effect on yield. Treatment time for the RF was chosen again to avoid visibly darkening the seeds.

## Results and Discussion

### Planting of corn seeds

**Figure 4:**
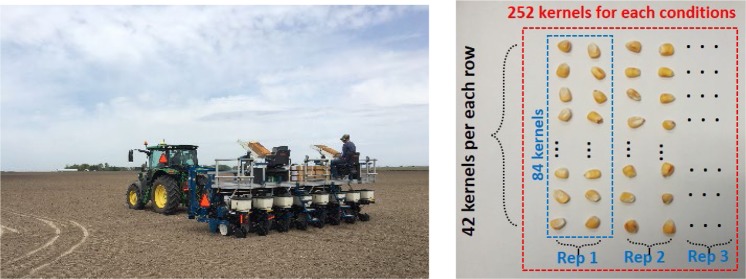
(**a**) Planting the plasma treated corn seeds. (**b**) Arrangement of the corn seeds during planting.

In April 2017, all plasma-treated corn seeds were prepared to plant in real fields as follows; (i) control, (ii) PAW treated, (iii) MAP treated, (iv) DBD plasma treated, (v) RF plasma treated and (vi) just vacuum treated. After the plasma treatment, Poncho/VOTiVO with Acceleron was applied at the recommended rate to all of the seeds. Poncho/VOTiVO with Acceleron is a standard treatment for corn seeds in the corn farming industry. All corn seeds were divided to 252 kernels (84 kernels × 3 replications) for each of 6 conditions and an entire 1512 seeds were planted in each of 6 locations: Macomb, Champaign, Windsor, Latham, Chrisman and Murrayville all in central Illinois. A planting machine with installed GPS was used to plant the seeds to an exact position with a uniform spacing as shown in Fig. 4a. At the start point of each row, a location marker was installed in the ground to distinguish each sample. The scheme of planting is also shown in Fig. 4b. In this experiment, we used a total of 9072 kernels.

### Growing and harvest

After planting the corn seeds in late April 2017, there was a series of weather events including heavy rain, flooding and cold temperatures. Later in the summer some plots were damaged through wind storms causing some stalks to lodge during very dry weather conditions near the harvest season. While the total average rainfall was in the normal range, the weather and water condition had unique features which could be responsible for the results. Harvesting was done in October 2017 by a 4 row combine machine with installed computer system to collect the yield information simultaneously during harvesting. This allowed instant calculation of the yield in units of bushels/acre.

### Data analysis

After the planted corn seeds were harvested, data was extracted as yield per condition in each location. Before making the histograms, any raw data points over 2σ from the average were discarded. These cases were clearly outliers from major impairments having nothing to do with the seed treatments (such as many days of standing water after a heavy rainfall). Statistically, these outliers could be considered as causing a depressed growth rate caused by nature (flood or wind damage to of the field), not originated from treatment effect. Also, missing data representing planting or harvest mistakes was not included. The revised average and standard deviation were calculated from the remaining data and six different histograms distinguished by location were obtained which reflected the plasma treatment effect on the corn as shown in Fig. 2. Error bars and the uncertainties shown are for one standard deviation.

**Figure 5:**
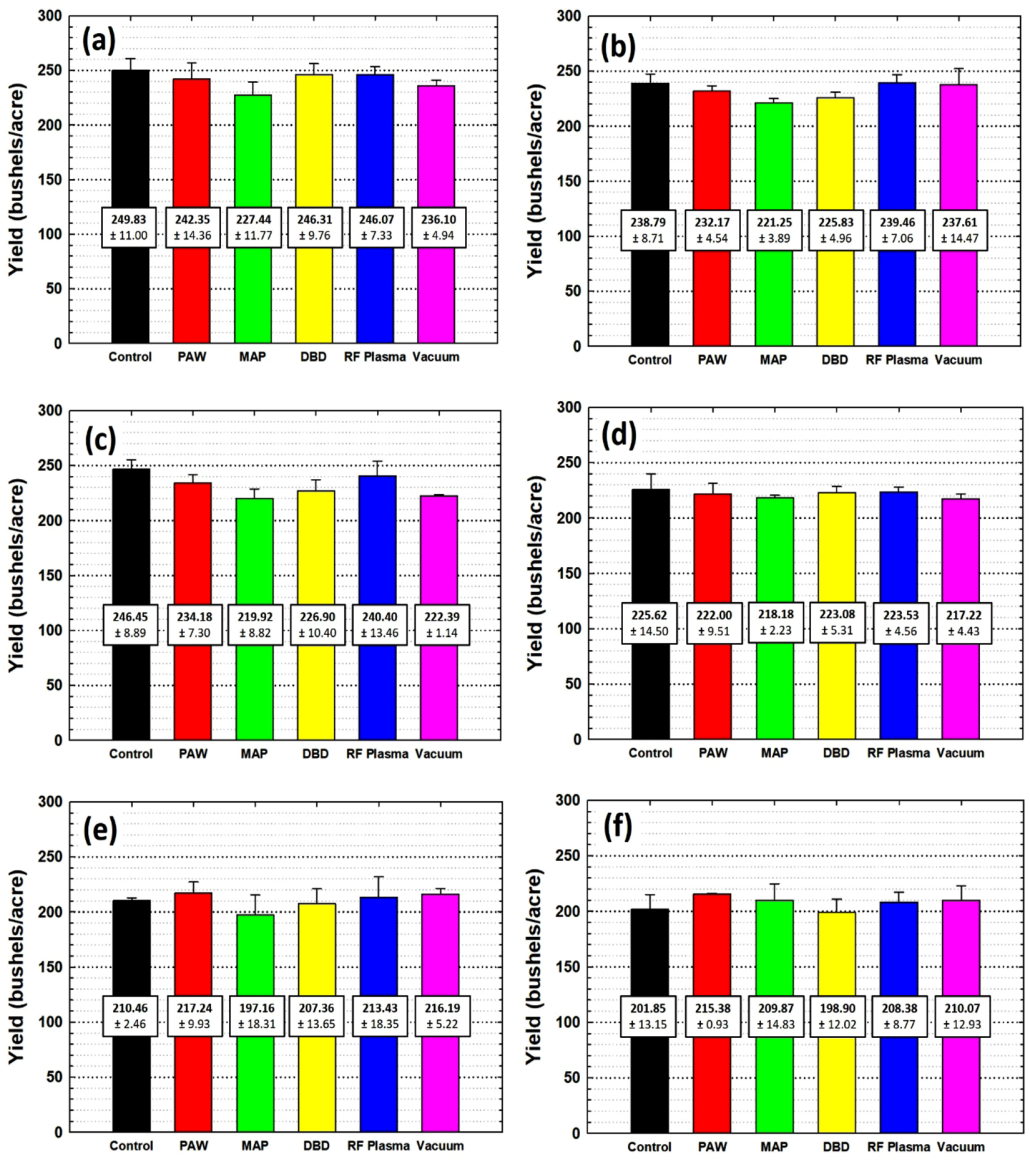
Harvested yield of corn in bushels/acre for (**a**) Macomb, (**b**) Champaign, (**c**) Windsor, (**d**) Latham, (**e**) Chrisman and (**f**) Murrayville locations, all in Illinois. Inset numbers are the average yield and standard deviation.

The yield results for Macomb, Champaign, Windsor and Lantham show a similar tendency according to each treatment variable as shown in Fig. 5a–d. In each of these fields the control samples had the best yields. However, the differences are small and all of the yields fall within one standard deviation of each other. At the same time, there is consistent increase in yield of the RF plasma treated samples compared to exposure to vacuum alone. Remember that both of these seed cohorts were exposed to vacuum for the same length of time. Therefore, the RF plasma treatment can be considered to have some positive effect in increasing corn growth rate compared to simply stressing and dehydrating the corn by exposure to vacuum. These results are similar to those shown in Volin *et al*.^8^ where SEMs of the seed surface after vacuum and RF plasma exposure show morphology changes which alter water take-up rates. The results from Chrisman and Murrayville (Fig. 5d,e) had significantly lower overall yields and less variation from one treatment to another.

**Figure 6:**
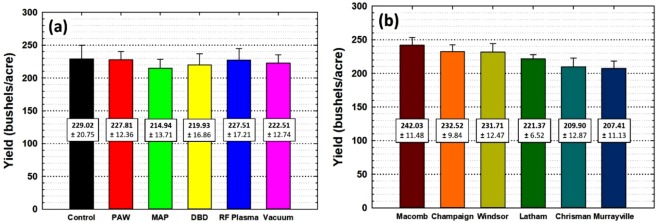
(**a**) Yields depends on plasma treatment variables and (**b**) on locations.

The data also shows that MAP treated samples recorded the lowest yield overall, and was the lowest in each field. For example in Champaign (Fig. 5b) the yield dropped from 238.8 +/− 8.7 to 221.3 +/− 3.9 bushels/acre. According to the literature, corn dried at 50 °C could be damaged^20^. Since the MAP apparatus generates plasma with a gas temperature of 600 °C or higher^16^, it is possible that the seeds were heated above 50 C resulting in a lower yield. Even though the corn seeds were far enough (2 cm) from the end of plasma region during the treatment to avoid burning or blackening, the temperature of the corn seeds could easily have exceeded the damage threshold. These results were the same for almost every location except the location in Murrayville (Fig. 5f). When we plot the histogram taking into account only treatment types regardless of location as shown in Fig. 6a, it supports the hypothesis that the thermal effect on the corn seeds caused a lower yield. The second lowest performer was the DBD plasma which also warmed the seeds during treatment though not nearly as much.

Figure 6b shows the overall yield for each location. The average yield in Chrisman and Murrayville were 209.90 +/− 12.87 and 207.41 +/− 11.13 bushels/acre respectively which is poor compared to other locations with total average yield of 231.91 +/− 5.04 bushels/acre calculated from the other four locations. These results could mean that unsteady weather conditions in this location affected the yield beyond the particular treatment of the corn seeds. However, it should be noted that the corn, which is assumed to have grown in unsteady weather in Chrisman and Murrayville, had a relatively higher average yield for the PAW treated samples for those two fields compared to other variables shown in Fig. 5e,f respectively. This is one of the few cases where one of the treatments resulted in a higher yield than the control, although still within the standard deviations of the other measurements. Several studies have shown that PAW has more nitrogen content than plain water which can serve as ecological fertilizers to grow plants^12,13^. This could be considered as a contribution to corn growth when not enough nutrients are received in an unsteady climate, possibly leading to relatively higher yields.

Nevertheless, it was expected that different plasma treatments would have a positive effect on the germination and growth of the corn seeds when planted in real fields based on past research results realized at the lab scale, however we could not identify any statistically significant differences through this experiment. One reason for this result is that the effect of plasma treatment on corn was minor compared to the variation in real field environmental effects such as weather and soil quality. Perhaps more importantly, the presence of Poncho/ VOTiVO with Acceleron being applied to all the seeds may have damped out other differences. This treatment is reported as a good fungicide for seed protection against soil and seed borne disease including fusarium, rhizoctonia and pythium and an insecticide for protection from early season pests, such as wireworm, seedcorn maggot, white grub, grape colapsis and black cutworm in crop growth^21^. This coating was applied because it is known to increase yields by at least 10% and in some cases protect a crop from catastrophic loss. Virtually all commercial corn planted in the US Midwest has either this or a similar coating applied. While a test with no additional biological treatment on the corn seeds would be a clearer way to determine the plasma treatment effect, it would not lead to commercial use unless the plasma treatment was effective after the corn was coated as in the results shown here.

## Conclusion

In this study, an extensive experiment was conducted to investigate if plasma treating corn seeds could have a positive effect when planted in real fields under commercial conditions similar to those successful results obtained in lab environments where early germination or fast growing rates are shown. The yield obtained for RF vacuum plasma treated corn shows relatively higher values than other conditions, but not as high as the control. PAW treatment applied with the Poncho/VOTiVO seed treatment had some positive effect on yield in locations with an unsteady natural environment. However, there are no statistically significant results overall. Commercial hybrid corn seeds planted in the US Midwest already have near-100% germination rates and have been engineered and selected to reach remarkable yield figures. Plasma treatments may best be applied to crops where such improvements in germination rates and yields have not already been achieved through other means.
